# Influence of Molecular Structure and Material Properties on the Output Performance of Liquid–Solid Triboelectric Nanogenerators

**DOI:** 10.3390/mi14101825

**Published:** 2023-09-24

**Authors:** Ziyun Ling, Fang Lin, Xili Huang, Hongchen Pang, Qianxi Zhang, Cheng Zhang, Xiaoning Li, Xianzhang Wang, Xinxiang Pan

**Affiliations:** 1 College of Naval Architecture and Shipping, Guangdong Ocean University, Zhanjiang 524088, China; 2112106011@stu.gdou.edu.cn (Z.L.); linfang@gdou.edu.cn (F.L.); panxx@gdou.edu.cn (X.P.); 2 College of Electronics and Information Engineering, Guangdong Ocean University, Zhanjiang 524088, China; 2112010010@stu.gdou.edu.cn; 3 College of Mechanical Engineering, Guangdong Ocean University, Zhanjiang 524088, China; neomailphc@gdou.edu.cn; 4 College of Ocean Engineering and Energy, Guangdong Ocean University, Zhanjiang 524088, China; zhangqx@gdou.edu.cn (Q.Z.); hustquick@gdou.edu.cn (C.Z.); xnli@gdou.edu.cn (X.L.)

**Keywords:** molecular structure, material properties, output performance, LS-TENG

## Abstract

With the advantages of superior wear resistance, mechanical durability, and stability, the liquid–solid triboelectric nanogenerator (LS-TENG) has been attracting much attention in the field of energy harvesting and self-powered sensors. However, most of the studies on LS-TENG focused on device innovations, changes in solid materials, and the effect of solid properties on output performance, and there is a lack of studies on liquids, especially at the molecular level. A U-tube LS-TENG was assembled to conduct experiments, whereby the effects of molecular structures, including molecular composition, carbon chain length, functional groups and material properties on the output performance were investigated. The deuterium replacing hydrogen and the atomic compositions could not achieve the enhancement of the output performance. Whether the chemical functional groups improve the output performance of LS-TENG depends on the mating solid material. Hydroxyl and cyanogenic groups can improve the output performance for the FEP case, while amide and cyanogenic groups can improve the output performance for the PTFE case. The order of output performances for functional groups of four groups of liquids with both FEP and PTFE materials is also obtained. It was also found that the dielectric constant is not positively correlated with the output performance. The results of this study might provide a reference for the deeper study and application of LS-TENG.

## 1. Introduction

Since Wang’s group proposed a triboelectric nanogenerator (TENG) in 2012, we have witnessed a growing academic interest in TENG owing to its outstanding performance in harvesting energy from the ambient environment [1,2,3]. The great efficiency of TENG in converting mechanical vibration with low frequency (typically < 10 Hz) into electricity has been evaluated in a number of studies [4,5,6]. The primary basis of TENG power generation is the transmission of electrons at the contact surface of two triboelectric materials [7]. There are several applications for TENG in solid materials, and numerous studies have demonstrated its high production and conversion rate [8,9,10,11]. Other than solid-solid TENG, recent studies have found that there is also electron transfer at the contact surface of liquid–solid materials [12]. Liquid materials are characterized by free flow and free deformation. As a result, compared to solid-solid materials, liquid materials can achieve a greater contact area and a higher contact efficiency at the liquid–solid interface [13]. Therefore, the liquid–solid interface has a potentially larger output power density than the solid-solid interface. Various liquid–solid TENGs (LS-TENG) have been invented and applied to water wave energy harvesting devices [14,15], medical health monitoring [16], motion sensors [17,18], etc. Additionally, LS-TENG is anticipated to perform better over a longer period of time due to the decreased abrasion at the liquid–solid interface compared to the solid-solid interface [1,14].

It is now widely accepted that increasing the density of the transferred charges at the liquid–solid interface could improve LS-TENG output. By modifying the surface or altering the characteristics of solid materials, researchers have been able to enhance the power output of LS-TENG [19,20,21,22,23]. Additionally, researchers have suggested a few solid-liquid combinations to improve output performance [7]. Besides, some studies have found that some material properties of liquids also affect output performance. Wang et al. [17] and Pan et al. [24] proposed the relationship between output and polarity/dielectric constant/contact angle. Shen et al. [25] proposed the influence of temperature on the performance of solid-liquid TENG. Besides this, a number of researchers are also exploring the mechanism of chemical functional groups to triboelectric initiation, especially for the liquid–solid cases. It has been found that surface modification with various functional groups can enhance the transmission of electrons between solids and liquids, resulting in maximization of the output performance of TENG [26,27,28]. Since chemical functional groups exist in chemical liquids, we studied the effect of functional groups on output performance by altering the type of liquid in LS-TENG. This is an optional method to improve the output performance besides surface modification of solid materials.

In this work, we assembled a simple U-tube LS-TENG and then applied various liquids for LS-TENG experiments to investigate the effects of the molecular structure of the chemical liquids and the intrinsic properties of the liquids on the output performance of the LS-TENG. From the perspective of molecular structure, not only the influence of the functional groups but also the isotopic substitution of chemical molecules and the influence of molecular chain length on the output performance are investigated. In addition, the effect of the dielectric constant on the output performance is analyzed.

## 2. Materials and Methods

### 2.1. Materials

Fluorinated ethylene propylene (FEP), Polytetrafluoro ethylene (PTFE), PVC electrical insulating tape and conductive enameled copper wire and foil were purchased from Alibaba’s e-commerce platform. Methanol, Ethanol, N-propanol, Ethylene glycol, Glycerol, Formamide, Acetamide, Propionamide, Malonamide, Formic acid, Oxalic acid, Pentane-1,3,5-tricarboxylic acid, Acetic acid, Benzonitrile, Acetonitrile, Succinonitrile, Deuterium oxide (D_2_O), Hydrogen peroxide (H_2_O_2_) were acquired from Shanghai Macklin Biochemical Co, Ltd. (Shanghai, China). Some liquids with specified concentrations in the experiment need to be prepared in advance. The concentrations of glycerol and H_2_O_2_ were set to 50% and 35%, respectively. Some of the other liquids purchased are solids that need to be prepared as 0.1 mol/L solutions. In order to completely rub the liquid and solid materials of the U-tube TENG to generate electricity, an electric rocker arm was used to shake the U-tube.

### 2.2. Fabrication of U-Tube L-S TENG

The U-tube LS-TENG, consistent with the device in [24], was assembled and used to conduct experiments. We chose a U-shaped tube with a thickness of 1.0 mm and an inner diameter of 6.0 mm (I.D. = 6.0 mm) to construct the U-tube TENG. Two conductive copper foils (50 µm thick) with a length of 6 cm (L = 6.0 cm) and a gap length of 8 cm (L_gap_ = 8.0 cm) were glued to the two columns of the U-tube TENG. During the pasting process, conductive copper wires that have been scraped off the paint were pasted between the U-tube wall and the copper foil. Then, we made the U-tube TENG for the experiment. In the experiment, we sequentially filled the liquid purchased above into the U-tube in a volume of 5.0 mL (V_liquid_ = 5.0 mL), and the liquid level was located in the center of the two Cu tapes.

A Hitachi SU8010 field-emission SEM (Hitachi High-Tech, Shanghai, China) was used to measure the inner surface morphology of the FEP and PTFE tubes. A mechanical rocker arm with adjustable swing frequency was used to swing the U-tube to achieve power generation. We used programmable electrometers (Keithley 6514 Electrostatic Meter, Tektronix, Shanghai, China) to test the open-circuit voltage (V_oc_), short-circuit current (I_sc_), and transferred charge (Q). The software platform was constructed based on LabView, which can realize real-time data acquisition and storage with a computer. In addition, some of the measured data were filtered. The FFT low pass filter (LPF) in the origin software was used to perform the data filtering. The LPF passes through low-frequency signals while blocking high-frequency signals so that the wave shape can be seen obviously. The filtering parameters are considered based on the wave shape.

## 3. Results and Discussion

### 3.1. Structure and Working Principle of the U-Tube TENG

**Figure 1:**
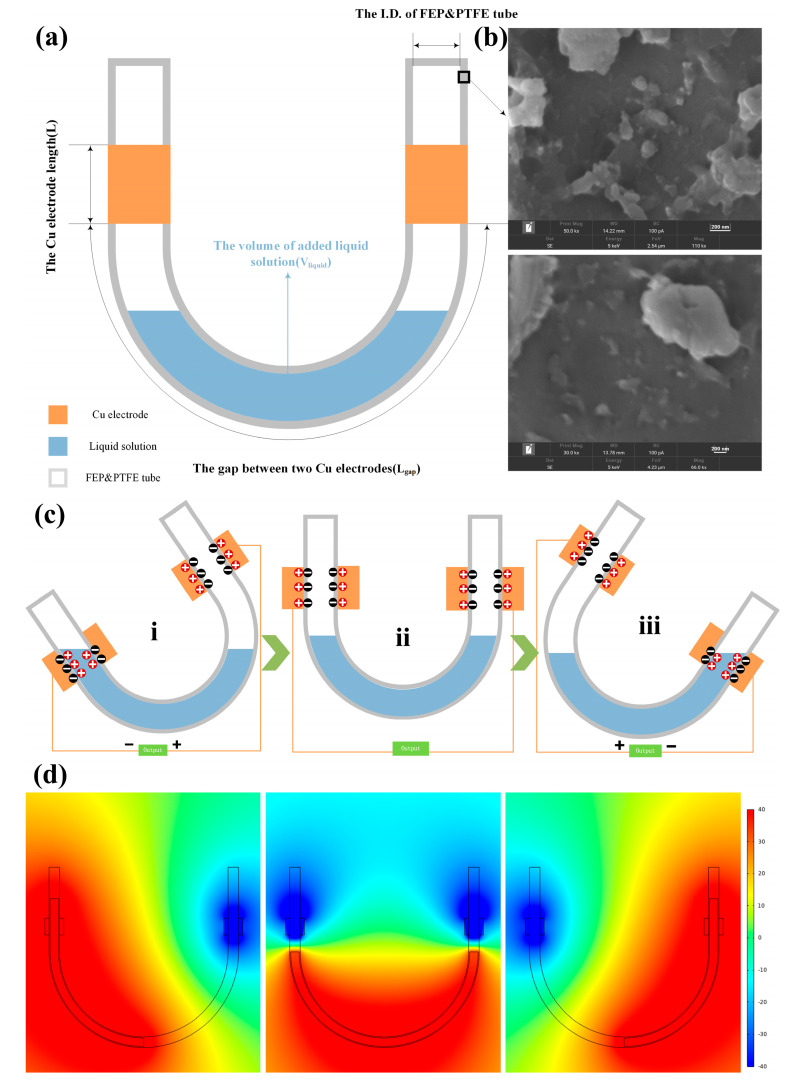
Structure design and working mechanism of the U-tube TENG. (**a**) Schematic illustration of the functional components of the U-tube TENG. (**b**) Characterizations of the inner surface of the solid materials, FEP and PTFE, used in the fabrication of the U-tube TENG. (**c**) Simulated potential distribution which was divided into three parts (i–iii) and (**d**) working principle (between the solid surface and liquid, simulated by COMSOL software) under different U-tube TENG positions.

As illustrated in Figure 1a, a U-tube liquid-fluorinated ethylene propylene (FEP)&poly tetrafluoroethylene (PTFE)-type TENG was assembled with three parts: FEP&PTFE U-tube, Cu electrode, and liquid solution. FEP and PTFE were chosen as the solid parts because these are excellent electronegative materials with pure carbon-fluorine structures. The SEM images in Figure 1b show the inner surface of the FEP and PTFE U-tube possessing a coarse nanostructure, which benefits the contact between the flowing liquid and the inner surfaces, and the free flow of the liquid also increases the contact area between the two materials, thus optimizing the output performance of the TENG. According to Pan’s study, the highest output was obtained with an inner diameter of 6 mm, a copper electrode length of 6 cm, a gap length of 8 cm between the two electrodes, and a liquid content of 5 mL for the experiment [24]. Given this, this set of parameters is also used for the experiments in this study. 

The liquid solution flowing in the inner surface of the U-tube is the vital factor for inducing the separation of the electrons and the positive charges in the solid and liquid solution, respectively. To theoretically predict the distribution of the electrical potential (i.e., the open-circuit voltage, V_oc_) between the two electrodes affixed to the two sides of the U-tube, COMSOL software (COMSOL Multiphysics 5.5) that employs the finite element method (FEM) was implemented to model the U-tube TENG. Based on the flowing process of the positively charged liquid, the working mechanism of the U-tube TENG and the simulation results of COMSOL were exhibited in Figure 1d. The working mechanism of the U-tube TENG is based on the freestanding model, so the whole power generation process includes three major steps. In the first step, the positive charges in the liquid equaled that of the contacted FEP surface when the freestanding positively charged liquid approached the left side of the FEP U-tube, resulting in a very less charge generated on the surrounding left Cu electrode. Meanwhile, there is no liquid balancing the negative charge of the right U-tube solid part, leading to an obvious accumulation of the positive charges on the right copper electrode. Accordingly, a high electric potential was generated on the right electrode. As the U-tube gradually swings to the right, the liquid covering the left electrode decreases, and the liquid near the right electrode increases. Thus, the flow of positive charge from right to left causes a gradual increase in current in the direction of right-to-left and maximums at position (ii). However, the potential difference between the two electrodes is zero at position (ii). Then, the U-tube further swings to the right so that the positively charged liquid covers the right wall surrounded by the copper electrode of the U-tube. Hence, a high electric potential on the left electrode was generated in step (iii). Similarly, the flow of positive charge from left to right causes a reverse current when the U-tube gradually swings to the left.

### 3.2. Effects of Molecular Structure on the TENG Output Performance

In order to investigate how the molecular structure of the liquids affects the output properties, the influence of the molecular structure, including isotopes and molecular composition, carbon chain lengths, and the number and type of different functional groups were analyzed.

#### 3.2.1. Effects of Isotopes and Molecular Composition

**Figure 2:**
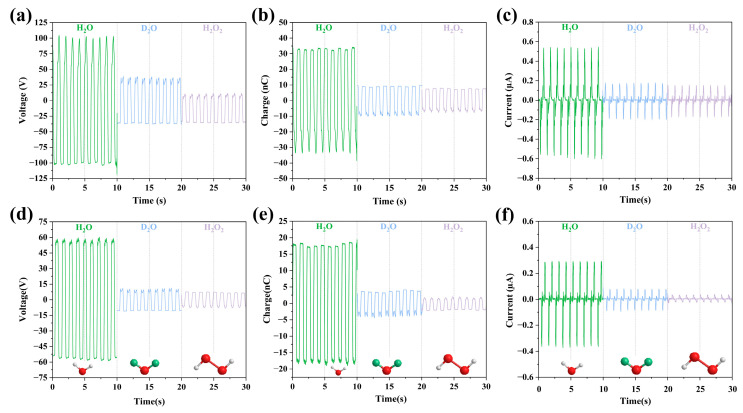
(**a**–**c**) The output parameters of H_2_O in FEP U-tube TENG tubes; (**d**–**f**) The output parameters of these three liquids in PTFE U-tube TENG tubes.

Experiments were conducted using pure water (distilled water), heavy water (deuterium oxide, D_2_O) and hydrogen peroxide (H_2_O_2_) at a concentration of 35% to investigate the effect of isotope and number of atoms on output performance. It is well known that heavy water is obtained by replacing the original hydrogen atoms with deuterium, an isotope of hydrogen. Hydrogen peroxide doubles the number of oxygen atomic compositions compared to pure water. For the 35% concentration, the molecular mass fraction of Oxygen increases to 91.5% from 89%. As can be seen from Figure 2, the output performances (voltage, charge and current) of FEP are higher than that of PTFE. Among the 3 cases, pure water obtains the optimal output performances, with a voltage of 105 V, charge of 33 n C and current of 0.6 μA. It is also evident that neither isotopic substitution nor molecular composition increases the output performance of TENG. Pure water has the best output performance in LS-TENG because its molecules are polar and easily interact with solid surfaces, resulting in friction charges. In addition, water molecules have a high surface tension and are easy to form water droplets, thus forming a stable charge on the solid surface.

#### 3.2.2. Effect of Carbon Chain Length

**Figure 3:**
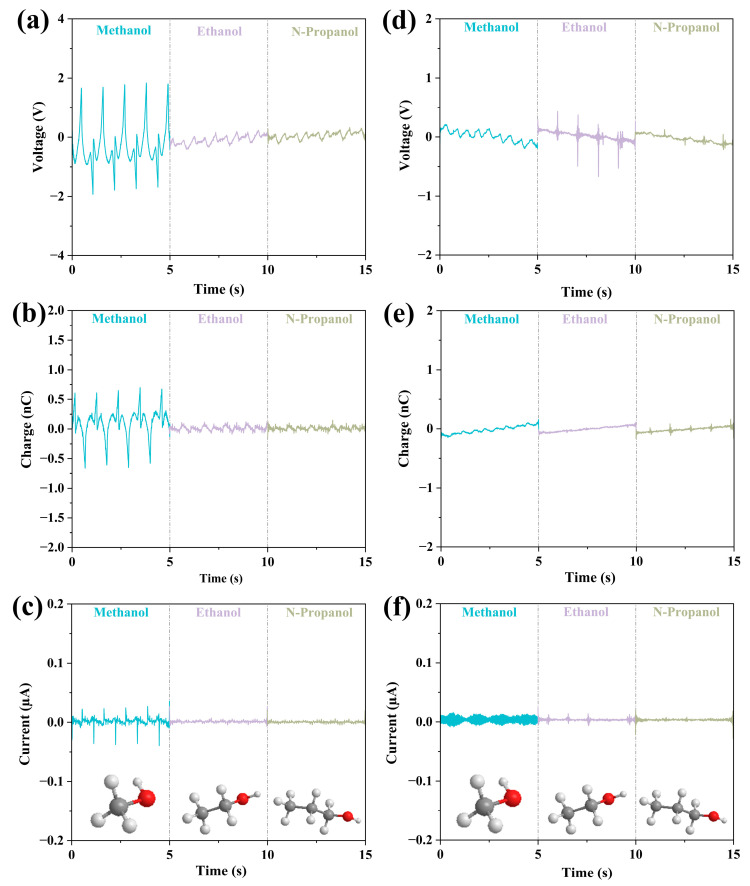
The data for the first set of liquids for which the comparison of the effect of carbon chain length on output parameters of methanol, ethanol, and n-propanol. (**a**–**c**) are the output properties as well as the molecular structures of the three liquids in FEP U-tube TENG tubes, and (**d**–**f**) are the output properties as well as the molecular structures of the three liquids in PTFE U-tube TENG tubes.

For the study of the effect of carbon chain length of chemical liquids on the output performance of LS-TENG, two groups of liquids were selected for the analysis. Firstly, comparing the three liquids, methanol, ethanol, and n-propanol, the chain lengths gradually increase from their molecular structure diagrams. As can be seen from Figure 3, again, the output performances of FEP are higher than that of PTFE. For the FEP case, as the carbon chain length increases, the output performances decrease. For the PTFE case, carbon chain length only has a negligible effect on the output performance.

**Figure 4:**
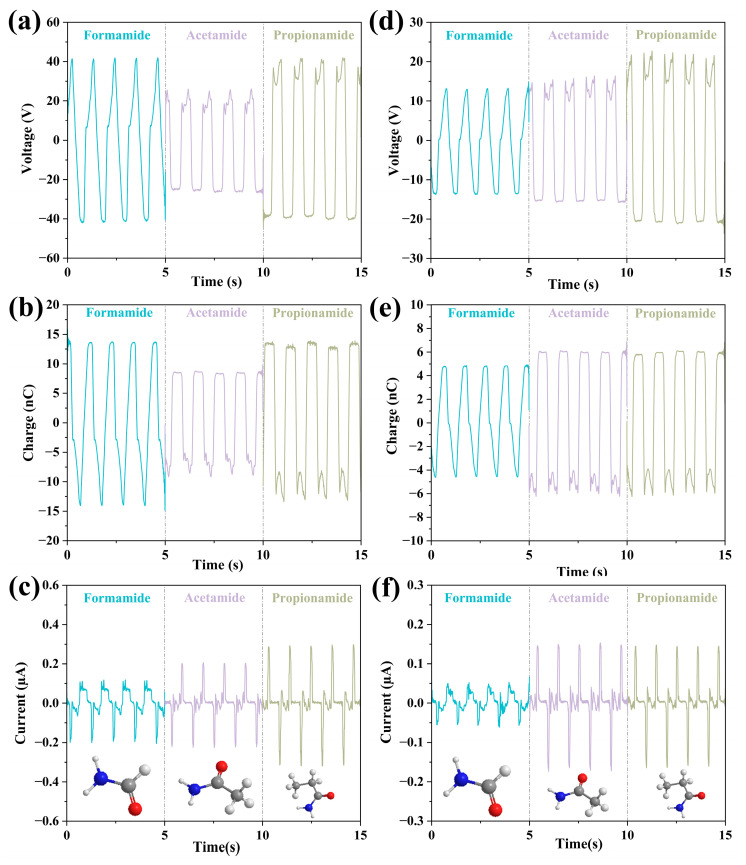
The data for the second set of liquids for which the comparison of the effect of chain length on output parameters of formamide, acetamide, and propionamide. (**a**–**c**) The output properties as well as the molecular structures of the three liquids in FEP U-tube TENG tubes; (**d**–**f**) The output properties as well as the molecular structures of the three liquids in PTFE U-tube TENG tubes.

The second group of liquids (formamide, acetamide and propionamide) has the same acylamino with different carbon chain lengths. As can be seen from Figure 4, the situation is slightly different. For the FEP case, as the carbon chain length increases, voltage and charge decrease then increase, i.e., these values of acetamide are lower than that of formamide and propionamide while current increases. For the PTFE case, as the carbon chain length increases, the output performances increase. 

The reason why the carbon chain length has a significant effect on the output performance of FEP but not the PTFE is discussed. For liquid friction materials with short carbon chain lengths, their molecular chains are easy to be oriented on the surface of FEP, so it is easy to form a stable charge and generate a large electrical energy output, while for those with long carbon chain lengths, its molecular chains are not easily oriented on the surface of FEP, so it is not easy to form a stable charge and generate a small electrical energy output. For PTFE, due to its low surface energy (or small molecular force), the carbon chain length of the liquid material has little effect on its power generation performance.

However, the experimental results obtained from the two groups show a different trend. For alcohol liquids, the hydroxyl group (-OH) in its molecular structure can hydrogen bond with the surface of PTFE, which may affect the output performance of the TENG device to some extent. However, this interaction may not change significantly when the alcohol carbon chain length is changed, so there is no obvious effect on the output performance of the TENG device. For amide-based liquids, the amide group (-CONH-) in its molecular structure can strongly interact with the surface of PTFE, and this interaction may have a significant impact on the output performance of the TENG device. This interaction may change when the amido-based carbon chain length is changed, thus affecting the output performance of the TENG device. As a result, alcohols and amido-based liquids may interact with PTFE in different ways, resulting in their carbon chain lengths having different effects on the output performance of the TENG device. It might be concluded that the effect of chain length on the output properties is determined by the liquid type and the mating solid material. 

#### 3.2.3. Effect of the Number of Different Functional Groups 

It has been found that for solid materials, i.e., solid polymers, the presence of functional groups can have an impact on the output performance of liquid–solid mode TENGs. The functional groups carried by the polymers can enhance the electron transfer and ion transfer during the contact initiation process, which can affect the output performance [26,29]. Inspired by the study of functional groups in solid materials, our study focuses on the effect of different functional groups carried by liquid materials on liquid–solid TENG. We analyze the effect of the number of functional groups in terms of four functional groups: hydroxyl, carboxyl, cyano group, and amide functional groups on the output properties of LS- TENG. 

**Figure 5:**
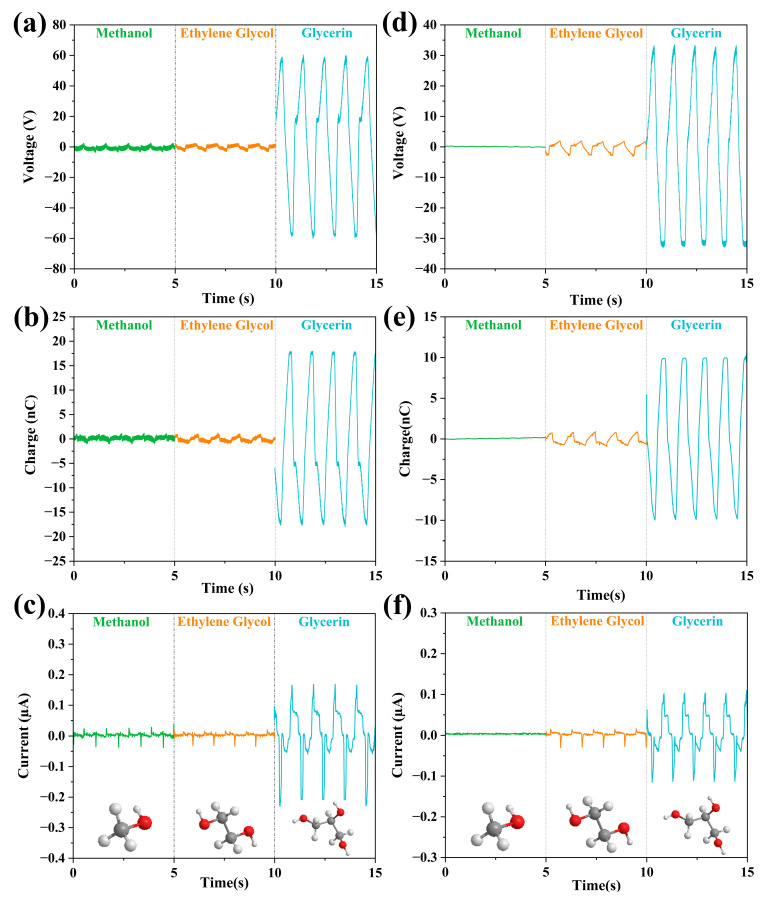
Molecular structure and comparison of output properties of hydroxyl liquids, where the bottom of each column shows the molecular structure of the liquid. (**a**–**c**) The change of the output parameters (V_oc_, Q, I_sc_) of the three liquids in the FEP U-tube TENG tube; (**d**–**f**) The change of the output parameters (V_oc_, Q, I_sc_) of the three liquids in the PTFE U-tube TENG tube.

Firstly, the hydroxyl functional groups are analyzed. We chose three liquids with the number of hydroxyl groups increasing from 1 to 3: methanol, ethylene glycol, and propanetriol, with a concentration of 50%. As shown in Figure 5, it is clearly seen that propanetriol with three hydroxyl groups is the best output among the three liquids. For the FEP case, the 50% concentration of propanetriol could reach a voltage of 60 V, a charge of 18 nC, and a current of 0.33 μA. The output performances of methanol and ethylene glycol just have little differences, with the latter being a little higher. For the PTFE case, as the carbon chain length increases, the output performances increase. Thus, an increase in the number of hydroxyl groups can improve the output performance of LS-TENG.

**Figure 6:**
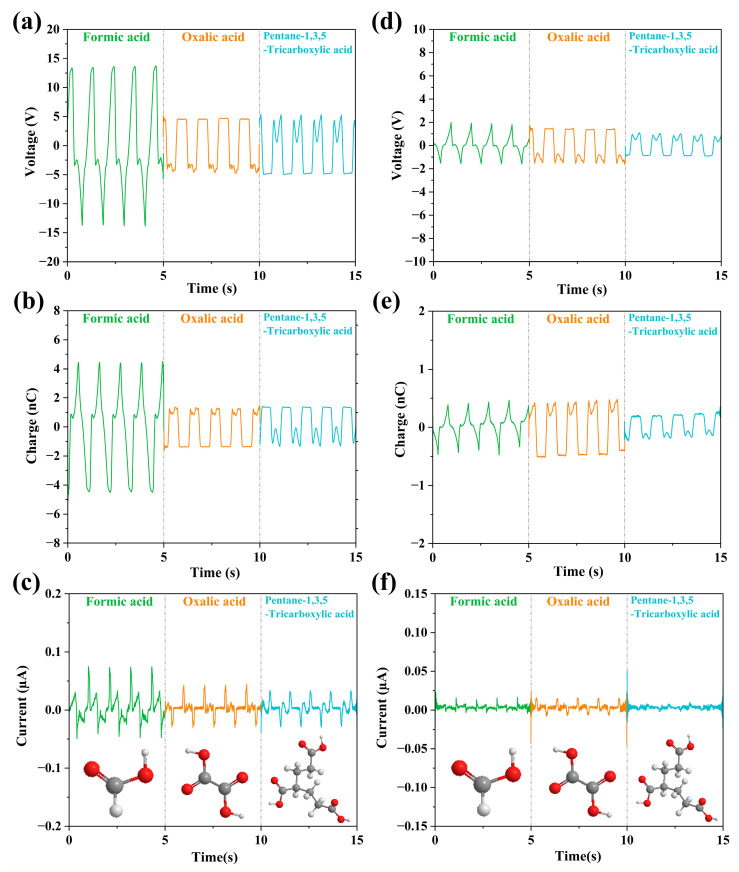
Molecular structure and comparison of output parameters of carboxylic liquids, where the bottom of each column shows the molecular structure of the liquid. (**a**–**c**) The change of the output parameters (V_oc_, Q, I_sc_) of the three liquids in the FEP U-tube TENG tube; (**d**–**f**) The change of the output parameters (V_oc_, Q, I_sc_) of the three liquids in the PTFE U-tube TENG tube.

Second, we carried out an analysis of the carboxylic group of liquids, which consisted of Formic acid, Oxalic acid, and Pentane-1,3,5-Tricarboxylic acid, while the number of carboxylic groups was increased from 1 to 3. As shown in Figure 6, for the FEP case, the output values of Formic acid are the highest (voltage of 15 V, charge of 4.8 n C, and current of 0.2 μA.), while those of Oxalic acid are the lowest (voltage of 5.8 V, charge of 1.6 n C, and current of 0.17 μA.) among these three liquids. Oxalic acid and Pentane-1,3,5-Tricarboxylic acid only have a little difference. For the PTFE case, all the values of voltage, current and charge also have neglective differences.

In LS-TENG, carboxyl groups can affect the wettability and adhesion of liquid on the solid surface through electrostatic interaction with the solid surface, thus affecting the power generation output performance. When the carboxyl group content is higher, the acidity of the liquid material is enhanced, and its wettability and adhesion on the solid surface are increased. This is beneficial to improve the power generation efficiency of the TENG, as the increase in wettability and adhesion can increase the charge density and friction of triboelectrification, thus generating more electrical energy. However, when the carboxyl content is too high, the viscosity of the liquid material will increase, making it less fluid, which may reduce the power generation efficiency of the TENG. In addition, excessive acidity may also negatively affect the mechanical properties and stability of LS-TENG, making it vulnerable to damage such as oxidation and corrosion.

**Figure 7:**
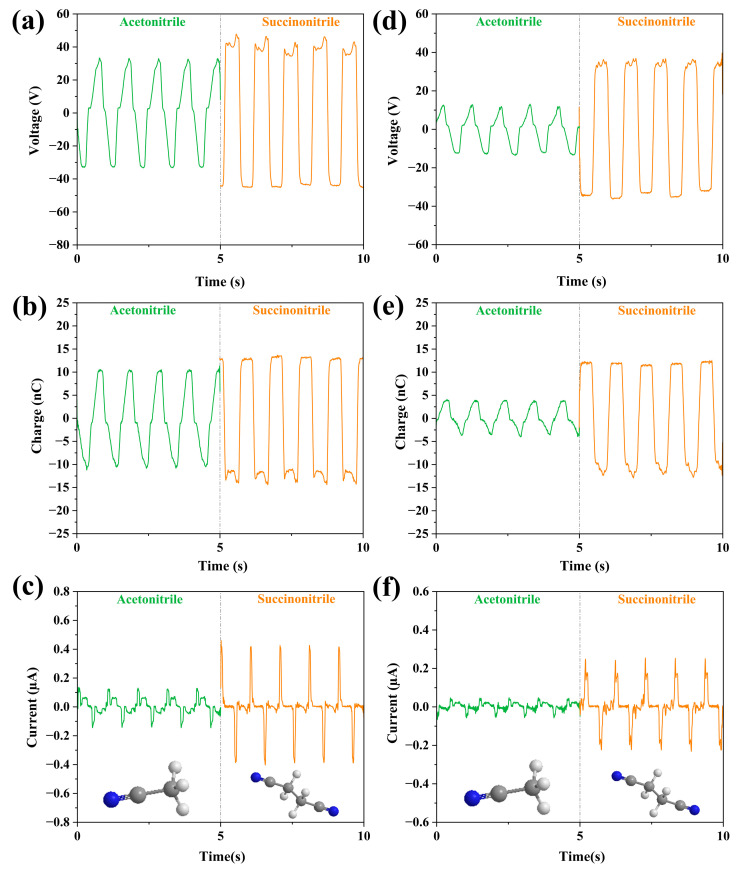
Molecular structure and comparison of output properties of cyanide liquids, where the bottom of each column shows the molecular structure of the liquid. (**a**–**c**) The change of the output parameters (V_oc_, Q, I_sc_) of the two liquids in the FEP U-tube TENG tube; (**d**–**f**) The change of the output parameters (V_oc_, Q, I_sc_) of the two liquids in the PTFE U-tube TENG tube.

Then, we studied and analyzed the cyano group of the liquids, including two liquids, acetonitrile and 0.1 mM succinonitrile. These two liquids have one cyano group and two cyano groups in their molecular structures, respectively. The output parameters of these two liquids are shown in Figure 7. The obtained results show that the output of succinonitrile with two cyanogenic groups in the molecule was better depending on whether the experiments were conducted in FEP or PTFE tubes. Especially in the PTFE tube, the effect of increasing the number of cyanogenic groups on the output performance is particularly significant. The V_oc_ of succinonitrile in PTFE tubes reaches 36 V, about twice the V_oc_ of acetonitrile. This suggests that the increase in the number of cyanogenic groups in the liquid molecular structure also enhances the output performance of L-S TENG. In LS-TENG, the cyano group in the liquids has no direct effect on the power output performance, but it can indirectly affect the power output performance by affecting the physical and chemical properties of the friction material. The presence of cyanide groups can increase the polarity and reactivity of liquid friction materials. This can cause the liquid friction material to generate more charge transfer when it comes into contact with other materials, thus enhancing the triboelectrification effect. The presence of a cyanide group can affect the adhesion and wettability of liquid friction materials. The increase of wettability and adhesion can increase the charge density and friction of triboelectrification, which helps to improve the output voltage and charge collection efficiency of LS-TENG.

**Figure 8:**
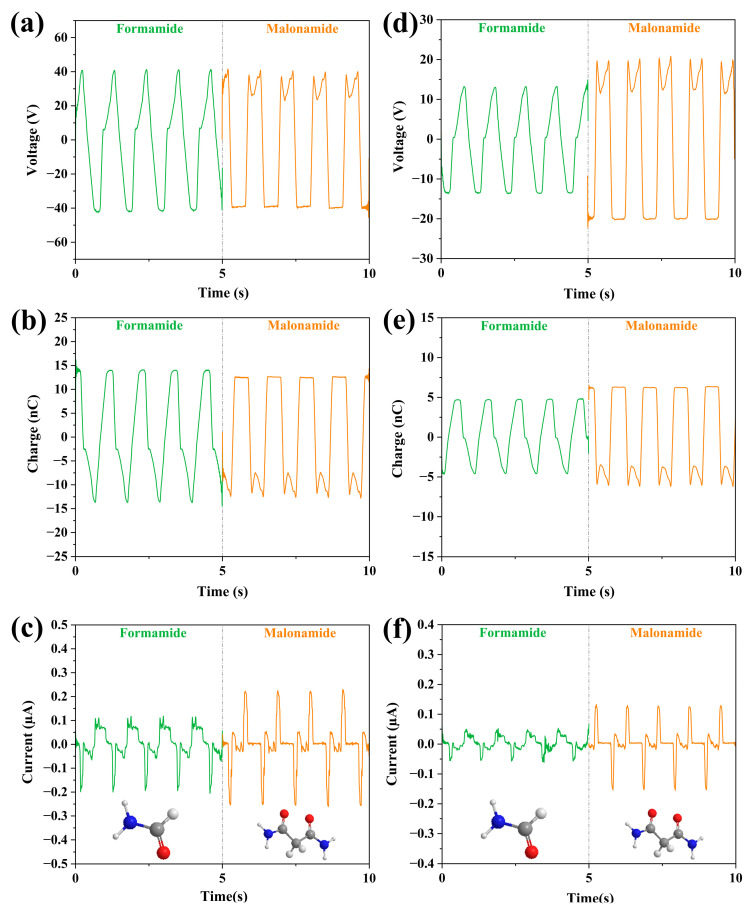
Molecular structure and comparison of output properties of amide-based liquids, where the bottom of each column shows the molecular structure of the liquid. (**a**–**c**) The change of the output parameters (V_oc_, Q, I_sc_) of the two liquids in the FEP U-tube TENG tube; (**d**–**f**) The change of the output parameters (V_oc_, Q, I_sc_) of the two liquids in the PTFE U-tube TENG tube.

Finally, we compared the amide-based liquids, propionamide and malonamide. As shown in Figure 8, only the value of charge increases a little, while the those of voltage and current decrease for the FEP case. All the values of the output parameters increase with the increasing number of amide groups for the PTFE case. Hence, it can be concluded that the increase in the number of amide groups has a negligible effect on the experimental results obtained for the FEP tubes. The increase in the number of amide groups could improve the output performance of the LS-TENG. 

**Figure 9:**
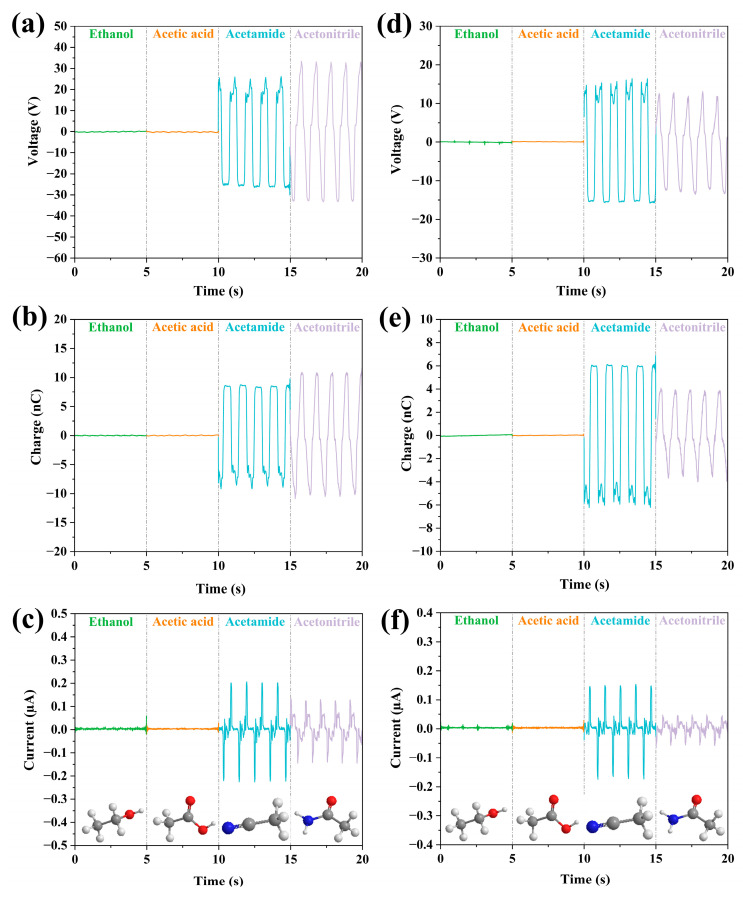
Output parameters of four liquids with different functional groups. (**a**–**c**) Experimental results of four liquids in FEP U-tube TENG; (**d**–**f**) Experimental results of four liquids in PTFE U-tube TENG.

In addition, a longitudinal comparison of functional groups was carried out. Ethanol, acetic acid, acetamide, and acetonitrile were selected to perform the experiments. The molecular structure of each liquid contains only one functional group, hydroxyl, carboxyl, amide, and cyanogenic functional groups. We could consider the functional groups -OH, -COOH, -CONH_2_, -CN as the representation of ethanol, acetic acid, acetamide, and acetonitrile, respectively, to investigate the output performance. As can be seen from Figure 9, both ethanol and acetic acid have lower values of output parameters compared with those of acetonitrile and acetamide. The order of output performances for functional groups of these four liquids is then obtained by comparing the experimental results. For the FEP case, the orders for these liquids are obtained: -OH < -COOH < -CONH_2_ < -CN for voltage and charge; -OH < -COOH < -CN < -CONH_2_ for current. For the PTFE case, the order for these liquids is: -OH < -COOH < -CN < -CONH_2_ for all the output parameters. It is found that the best output among the four liquids could be acetonitrile with amide group or cyanogenic with acetonitrile, depending on the mating solid material.

In this section, several experimental liquids were selected into groups and analyzed by functional groups. We investigated hydroxyl, carboxyl, cyanogenic, and amide-based liquids separately. We found that an increase in the number of hydroxyl, cyanogenic, and amide groups can enhance the output performance of TENG in liquid–solid mode, whereas an increase in the number of carboxyl groups decreases the output performance. Additionally, for a single longitudinal comparison of the different functional groups, the cyanogenic and amide groups are the functional group choices that provide better output properties.

### 3.3. Effects of Liquid Properties on the TENG Output Performance

In order to investigate the relationship between the liquid material properties and the output performance of the L-S TENG, more liquids were selected for the experiments. A total of 27 liquids were used for this exploratory experiment, and the values of the dielectric constants of these liquids were also found and collated. The results of the experiments on these liquids, i.e., the output parameters (V_oc_, I_sc_, Q) and the dielectric constant values, are listed in descending order of the dielectric constant values and are presented in Table S1 of the Supporting Information.

As reported, the voltage V for the dielectric-to-dielectric sliding-mode TENG can be estimated by Equation (1) [30]

(1) $$V=\sigma \omega \upsilon R\ln(\frac{l-\upsilon t}{l})$$

where σ is the tribo-charge surface density and ω, *l*, and *υ* are the dielectric width, dielectric length, and velocity, respectively. For the liquid-FEP& PTFE U-tube TENG with fixed parameters (ω, *l*, and *υ*), σ is the most important parameter for determining the output and is closely dependent on the inherent liquid properties

**Figure 10:**
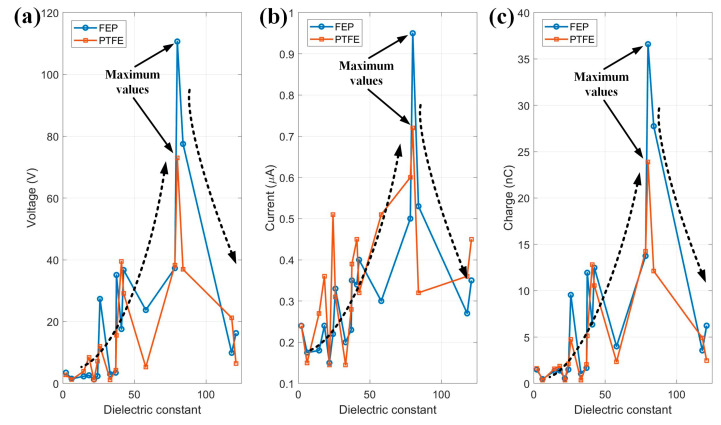
Relationship between the output performance of the U-tube L-S TENG and the dielectric constant of the liquid (**a**–**c**) Relationship between the values of the dielectric constants of the liquids involved and V_oc_, I_sc_, and Q.

In order to study the relationship between dielectric constant and output performance, several typical liquids are selected, and the experiment data of voltage, current and charge versus dielectric constant are plotted in Figure 10. Then, curves were fitted to show the trend clearly. As shown in Figure 10, the dotted black arrows are used to show the trend. All the values of voltage, current and charge increase gradually to a maximum value, then increase sharply with increasing values of dielectric constant, both in LS TENG with FEP as solid material and PTFE as solid material.

The dielectric constant of liquid reflects the electrical properties of the liquid medium, which depends on the molecular structure and molecular polarizability of the liquid. When the dielectric constant of the liquid increases, the polarizability of the liquid molecules increases, resulting in enhanced electrostatic interaction between the liquid and the solid surface. This makes it easier for charges on the surface of the solid to be attracted to the liquid molecules, increasing the amount of charge accumulated. At the same time, due to the increase in the polarizability of liquid molecules, the electric field intensity inside the liquid will also increase, which is conducive to the separation and transmission of electric charges. The combined effect of these factors makes the output voltage and current of the liquid–solid friction nanogenerator increase to some extent with the increase of the dielectric constant of the liquid. However, when the dielectric constant of the liquid continues to increase, the interaction between the liquid molecules may be enhanced, which complicates the electric field distribution inside the liquid, and the charge transmission is hindered. In addition, the high dielectric constant of the liquid may cause the charge to be strongly adsorbed between liquid molecules, making it difficult to transfer the charge from the liquid surface to the solid surface. The combined effect of these factors makes the output voltage and current of the LS-TENG decrease when the dielectric constant of the liquid continues to increase. Therefore, the output parameters of the LS-TENG show a trend of first increasing and then decreasing. Therefore, when designing and optimizing the TENG, it is necessary to carefully consider the structure, functional group content and other properties of the liquid material to achieve the best power generation output performance.

## 4. Conclusions

In this study, a simple liquid–solid U-tube TENG was assembled, and various types of liquids were applied to perform experiments to investigate the influence of the molecular structure and material properties of the liquid on the output performance of the LS-TENG. In addition, FEP and PTFE were selected for the mating solid materials, respectively. Firstly, at the atomic level, isotopic substitution, i.e., deuterium replacing hydro-gen, and the increase in the number of atomic compositions were not able to achieve the enhancement of the output performance. Subsequently, the effect of carbon chain length on the output performance was investigated. Then, the effect of chemical functional groups of the liquids on the output performance was studied. Whether the function groups of liquids improve the output performance depends on the type of mating solid material. Hydroxyl and cyanogenic groups in the liquids can improve the output performance for the FEP case, while amide and cyanogenic groups in liquids can improve the output performance for the PTFE case. Increasing the number of functional groups of the molecule was used to determine if it is beneficial to the output performances. The order of output performances for functional groups of four groups of liquids with FEP and PTFE materials is also obtained. Finally, the intrinsic material properties of the liquids were studied. It is found that the dielectric constant is not always positively correlated with the output performance. As the dielectric constant increases, the output parameters increase at a maximum value and then decrease.

This study investigated the effects on the output performance of LS-TENG from a chemical view, which is a little different from previous studies. The results might provide a reference for the deeper study and application. However, how the molecular arrangement and intermolecular affect the output performance is still unclear, which looks worthy of further investigation.

## Data Availability

The data presented in this study are available on request from the corresponding author.

## Supplementary Materials

The following supporting information can be downloaded at: https://www.mdpi.com/article/10.3390/mi14101825/s1, Table S1: Liquid and dielectric constant values used for liquid characterization.
