# Supplementary material for: Influence of Molecular Structure and Material Properties on the Output Performance of Liquid–Solid Triboelectric Nanogenerators
Source: Micromachines (Basel). 2023 Sep 24;14(10):1825. doi: 10.3390/mi14101825 (PMC10609343; doi:10.3390/mi14101825)
Supplement: Supplementary file 1 [file micromachines-14-01825-s001.zip › micromachines-2590452-supplementary.pdf]

micromachines

Supplementary Materials

Influence of Molecular Structure and Material Properties on the Output Performance of Liquid–Solid Triboelectric Nanogenerators

Ziyun Ling [1], Fang Lin [1], Xili Huang [2], Hongchen Pang [3], Qianxi Zhang [4], Cheng Zhang [4], Xiaoning Li [4], Xianzhang Wang [3,*] and Xinxiang Pan [1]

[1] College of Naval Architecture and Shipping, Guangdong Ocean University, Zhanjiang 524088, China; 2112106011@stu.gdou.edu.cn (Z.L.); linfang@gdou.edu.cn (F.L.); panxx@gdou.edu.cn (X.P.)
[2] College of Electronics and Information Engineering, Guangdong Ocean University, Zhanjiang 524088, China; 2112010010@stu.gdou.edu.cn
[3] College of Mechanical Engineering, Guangdong Ocean University, Zhanjiang 524088, China; neomailphc@gdou.edu.cn
[4] College of Ocean Engineering and Energy, Guangdong Ocean University, Zhanjiang 524088, China; zhangqx@gdou.edu.cn (Q.Z.); hustquick@gdou.edu.cn (C.Z.); xnli@gdou.edu.cn (X.L.)
* Correspondence: wangxianzhang@gdou.edu.cn

Table S1. Liquid and dielectric constant values used for liquid characterization.

Experimental Liquids	Dielectric Constant	Remark
Carbon tetrachloride	2.2	68°F
Acetic acid	6.2	68°F
Hexane	14.6	59°F
Isopropanol	18.3	None
Propanol	21.3	68°F
Ethyl Alcohol	24.3	77°F
Benzonitrile	26	68°F
Methanol	33.1	68°F
Ethylene Glycol	37	68°F
Acetonitrile	37.5	70°F
Acetamide	41	68°F
Glycerin	42.5	77°F
Formic acid	58	60°F
Deuterium oxide	78.3	77°F
Pure water	80	80°F
Formamide	84	68°F
Iodine	118	107°F
35% Hydrogen peroxide	121	None
